# Founder mutations and genotype-phenotype correlations in Meckel-Gruber syndrome and associated ciliopathies

**DOI:** 10.1186/2046-2530-1-18

**Published:** 2012-10-01

**Authors:** Katarzyna Szymanska, Ian Berry, Clare V Logan, Simon RR Cousins, Helen Lindsay, Hussain Jafri, Yasmin Raashid, Saghira Malik-Sharif, Bruce Castle, Mushtag Ahmed, Chris Bennett, Ruth Carlton, Colin A Johnson

**Affiliations:** 1 Section of Ophthalmology and Neurosciences, Leeds Institute of Molecular Medicine, St. James’s University Hospital, Leeds, UK; 2 Yorkshire Regional Genetics Service, St. James’s University Hospital, Leeds Teaching Hospitals NHS Trust, Leeds, UK; 3 GeneTech Lab, 46/1 Shadman Colony, Jail Road, Lahore, Pakistan; 4 Department of Obstetrics & Gynaecology, King Edward Medical College, Lahore, Pakistan; 5 Department of Clinical Genetics, Chapel Allerton Hospital, Leeds Teaching Hospitals NHS Trust, Leeds, UK; 6 Peninsula Clinical Genetics Service, Royal Devon and Exeter NHS Foundation Trust, Exeter, UK

**Keywords:** Meckel-Gruber syndrome, Genotype-phenotype, Founder mutation

## Abstract

**Background:**

Meckel-Gruber syndrome (MKS) is an autosomal recessive lethal condition that is a ciliopathy. MKS has marked phenotypic variability and genetic heterogeneity, with mutations in nine genes identified as causative to date.

**Methods:**

Families diagnosed with Meckel-Gruber syndrome were recruited for research studies following informed consent. DNA samples were analyzed by microsatellite genotyping and direct Sanger sequencing.

**Results:**

We now report the genetic analyses of 87 individuals from 49 consanguineous and 19 non-consanguineous families in an unselected cohort with reported MKS, or an associated severe ciliopathy in a kindred. Linkage and/or direct sequencing were prioritized for seven MKS genes (*MKS1*, *TMEM216*, *TMEM67/MKS3*, *RPGRIP1L*, *CC2D2A*, *CEP290* and *TMEM237*) selected on the basis of reported frequency of mutations or ease of analysis. We have identified biallelic mutations in 39 individuals, of which 13 mutations are novel and previously unreported. We also confirm general genotype-phenotype correlations.

**Conclusions:**

*TMEM67* was the most frequently mutated gene in this cohort, and we confirm two founder splice-site mutations (c.1546 + 1 G > A and c.870-2A > G) in families of Pakistani ethnic origin. In these families, we have also identified two separate founder mutations for *RPGRIP1L* (c. 1945 C > T p.R649X) and *CC2D2A* (c. 3540delA p.R1180SfsX6). Two missense mutations in *TMEM67* (c. 755 T > C p.M252T, and c. 1392 C > T p.R441C) are also probable founder mutations. These findings will contribute to improved genetic diagnosis and carrier testing for affected families, and imply the existence of further genetic heterogeneity in this syndrome.

## Background

MKS is an autosomal recessive lethal condition characterized by occipital meningoencephalocele, polycystic kidneys, postaxial polydactyly and ductal plate malformation of the liver. Other frequently observed features can include the Dandy-Walker malformation (or other posterior fossa defects), dextrocardia, bowing of long bones, cleft lip and/or palate, *situs inversus*, low set ears, microphthalmia and iris coloboma. To date, mutations in nine MKS genes are reported as causative (Table 1). The protein products of the nine MKS genes are all involved in the structure or function of either the ciliary basal body/transition zone or the axoneme of the primary cilia [1-5]. MKS is, therefore, the most severe inherited condition in a suite of similar conditions known as ciliopathies. Other conditions with ciliary involvement include Joubert syndrome, nephronophthisis, Bardet-Biedl syndrome, COACH syndrome and Senior-Løken syndrome. All of these syndromes are allelic at some loci, and share some phenotypic features. Primary cilia are ubiquitous organelles, which contribute to the multiorgan involvement in MKS and other ciliopathy phenotypes. 

**Table 1:** Genes mutated in Meckel-Gruber syndrome and related ciliopathies

**Locus**	**Chromosome location**	**Gene name**	**Protein name**	**Other ciliopathies**	**Key reference**	**Common mutation in population**	**Reference**
MKS1	17q22	*MKS1*	MKS1	BBS13	[1]	Finnish - c. 1408-35_1408-7del29	[1]
MKS2	11q13.1	*TMEM216*	TMEM216	JBTS2	[2]	Ashkenazi - p.R73L	[2]
	11q12.2	*TMEM138*	TMEM138	JBTS16	[3]		
MKS3	8q22.1	*TMEM67*	MECKELIN	JBTS6, NPHP11	[4]	Pakistani - c. 1575 + 1 G > A	[4]
MKS4	12q21.32	*CEP290*	CEP290	BBS14, JBTS5, LCA10, NPHP6, SLSN6	[5]		
MKS5	16q12.2	*RPGRIP1L*	RPGRIP1L	JBTS7, NPHP8	[6]	Mixed European -p.T615P	[11]
MKS6	4p15.33	*CC2D2A*	CC2D2A	JBTS9	[7]	Finnish - c. 1762 C > T	[7]
MKS7	3q22.1	*NPHP3*	NPHP3	NPHP3	[8]		
MKS8	12q24.31	*TCTN2*	TCTN2		[9]		
MKS9	17p11.2	*B9D1*	B9D1		[10]		

The nine genes reported to be mutated are listed, with key references indicated. Common founder mutations that have been identified previously are also indicated, with the ethnicity of the population studied and any relevant reference.

The genetic heterogeneity and phenotypic variability in MKS have hindered the development of an evidence-based strategy for genetic diagnosis. To facilitate the process of genetic diagnosis for families, the unequivocal identification of pathogenic variants, genotype-phenotype correlations and founder mutations in specific ethnic groups, therefore, has important clinical utility. To further define the allelic series of pathogenic mutations for seven of the nine known MKS genes, we have screened an unselected cohort of 87 separate individuals affected with MKS, from a total of 49 consanguineous families and 19 non-consanguineous. We report the identification of mutations in 26 consanguineous and 13 non-consanguineous families, and describe a total of 18 previously unreported mutations.

## Methods

### Patient ascertainment and research ethics statement

Blood samples and/or DNA samples from fetuses diagnosed with Meckel-Gruber or Meckel-like syndrome, unaffected siblings and parents were collected from UK centers. Adults were recruited to research studies after informed consent, with adherence to the Declaration of Helsinki ethical principles for medical research involving human subjects. Studies had favorable ethical approval from Leeds (East) Local Research Ethics Committee (study title “Molecular genetic investigations of autosomal recessive conditions”, REC reference number 08/H1306/85). DNA was extracted using QIAGEN (Crawley, West Sussex, UK) extraction kits following the manufacturer’s protocol by the Yorkshire Regional Genetics Service (http://www.leedsth.nhs.uk/sites/genetics/). Other DNA samples were obtained from referring clinicians or collaborators.

### Microsatellite genotyping and direct Sanger sequencing

DNA from affected individuals was genotyped for microsatellite markers that flanked seven of the nine known MKS genes (*MKS1*, *TMEM216*, *TMEM67/MKS3*, *RPGRIP1L*, *CC2D2A*, *CEP290* and *TMEM237*) at <1 cM genetic distance (Additional file 1: Table S1). Markers were PCR amplified using standard protocols, with the forward primer 5′ end-labelled with FAM (Sigma-Aldrich Ltd., Gillingham, Dorset, UK). Samples were run on an ABI3100 sequencer with ROX-500 (Applied Biosystems, Inc. Carlsbad, CA, USA) size standard. In samples from singleton or multiplex affected individuals of consanguineous origin, two homozygous markers indicated putative linkage to a locus under investigation, prioritizing the gene for subsequent sequencing. In non-consanguineous multiplex families, two or more affected individuals sharing haplotypes for flanking markers indicated putative linkage to a locus. DNA samples from singleton non-consanguineous samples were screened for the seven MKS genes described above. If linkage analysis (when performed) did not specifically exclude the involvement of the *MKS1*, *MKS2*, *MKS3* and *MKS6* loci, patients were then sequenced for the *MKS1*, *TMEM216*, *TMEM67* and *CC2D2A* genes. PCR primers were designed using Primer3 software (http://frodo.wi.mit.edu/primer3/) covering all coding exons and flanking intronic regions (Additional file 2: Table S2). A total of 188 coding exons were amplified by standard PCR protocols. PCR products were then purified using Exo-SAP (USB) following the manufacturer’s protocol. Bidirectional Sanger sequencing was performed using a “BigDyev3.0” sequencing kit (Applied Biosystems, Inc.) by standard protocols recommended by the manufacturer. Samples were run on a ABI3100 sequencer and analyzed using “SeqScape” and “Sequencing Analysis” software (both Applied Biosystems, Inc.).

### Analysis of mutations

The expected segregation of putative mutations was confirmed in families, whenever possible, and their absence was confirmed in databases of common benign variants (dbSNP http://www.ncbi.nlm.nih.gov/projects/SNP/, 1000 Genomes Project http://www.1000genomes.org/ and http://evs.gs.washington.edu/EVS/ as appropriate). The pathogenic potential of putative missense mutations was assessed by analysis with PolyPhen2 (http://genetics.bwh.harvard.edu/pph2/), or by manual comparison of CLUSTALX alignments of protein homologues to determine the phylogenetic conservation of mutated amino acid residues. We confirmed the absence of the mutant alleles in a panel of 96 DNA samples from ethnically-matched normal control individuals.

## Results

**Figure 1:**
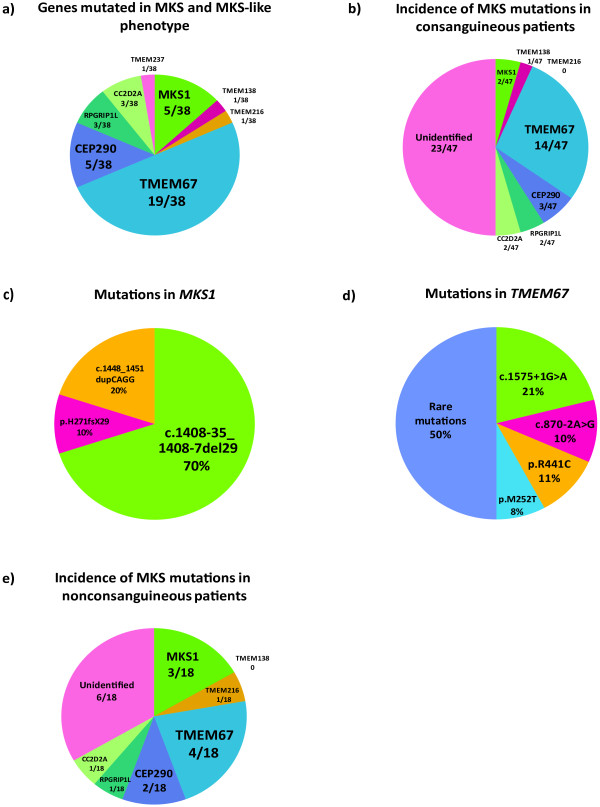
** Pie charts summarizing mutation analysis in MKS and MKS-like patients. a**) frequency of genes mutated in MKS and MKS-like phenotype; **b**) frequency of MKS genes mutations in consanguineous patients; **c**) common mutations in *MKS1*; **d**) common mutations in *TMEM67*; **e**) frequency of MKS genes mutations in non-consanguineous patients.

In this study we identified mutations in n = 38/68 (55.9%) families that were recruited to the study (Table 2). Out of all families with identified mutations, 19 (50%) had changes in *TMEM67* (Figure 1a), which highlights the prevalence of *TMEM67* mutations as a major cause of MKS. The second most commonly mutated genes were *CEP290* and *MKS1* (each n = 5/68 families; 3.2%). Mutations in *CC2D2A* and *RPGRIP1L* each had mutations in n = 3/68 families (7.9%). *TMEM216*, *TMEM138* and *TMEM237* each had mutations in only one family (2.6% each), confirming that these were uncommon causes of the MKS phenotype.

**Table 2:** Clinical data and sequencing results of consanguineous and non-consanguineous patients with MKS and MKS-like phenotypes

**Sample**		**Mutation**			**Phenotype**						
**Id**	**Ethnicity**	**Gene**	**Allele 1**	**Allele 2**	**OE**	**PK**	**PD**	**DPM**	**CLP**	**DWM**	**Other**
**Consanguineous**											
102 + 103 + 244 + 270	Pakistani	MKS1	c. 1448_1451dupCAGG	MKS1 c. 1448_1451dupCAGG	+	+	+		+		Short neck, low set ears, bilateral talipes, syndactyly, micropenis, *situs inversus*, congenital heart defect inc. dextrocardia, short femurs and short spindle-shaped tibiae, deformed tongue
264	Jordanian	MKS1	c. 1408-35_1408-6del30^*N*^	c.1408-35_1408-6del30^*N*^							diagnosed with MKS
42 + 43	Pakistani	TMEM138	c. A287G p.H96R	c. A287G p.H96R	+	+		+			
29A + 33A	Pakistani/Mirpuri	TMEM67	c. 1575 + 1 G > A	c. 1575 + 1 G > A	+	+	+	+			
70	Pakistani/Mirpuri	*TMEM67*	c. 1575 + 1 G > A	c. 1575 + 1 G > A		+				+	
76	Pakistani/Mirpuri	*TMEM67*	c. 1575 + 1 G > A	c. 1575 + 1 G > A	+	+					
77117	Pakistani	*TMEM67*	c. 1575 + 1 G > A	c. 1575 + 1 G > A							diagnosed with MKS
51	Pakistani/Mirpuri	*TMEM67*	c. 870-2A > G	c. 870-2A > G	+						
73	Pakistani/Mirpuri	*TMEM67*	c. 870-2A > G	c. 870-2A > G	+	+		+			
319	British	*TMEM67*	c. 1392 C > T p.R441C	c. 1392 C > T p.R441C		+		+			some dialation of pancreatic ducts, hydrocephalus, posterior fossa cyst
347	Pakistani	*TMEM67*	c. 1392 C > T p.R441C	c. 1392 C > T p.R441C							diagnosed with MKS
67FB	Pakistani	*TMEM67*	c. 647delA, p.E216fsX221	c. 647delA, p.E216fsX221	+	+		+	+		
P95	Pakistani	*TMEM67*	c. 1127A > C p.Q376P	c. 1127A > C p.Q376P	+	+		+			
125	Omani	*TMEM67*	c. 383_384delAC p.H128fsX140	c. 383_384delAC p.H128fsX140	+	+	+			+	
170	Turkish	*TMEM67*	c. 1674 + 1 G > A^*N*^	c. 1674 + 1 G > A^*N*^							diagnosed with MKS
205	Chinese	*TMEM67*	c. 1615 C > T p.R549C^*N*^	c. 1615 C > T p.R549C^*N*^		+		+			hypoplastic cerebellum, small fourth ventricle with large cisterna magna, small defect in superior aspect of occipital bone
C28	Pakistani	*TMEM67*	c. 274 G > A p.G92R^*N*^	c. 274 G > A p.G92R^*N*^							MTS, coloboma, mental retardation
39	Pakistani/Mirpuri	*CEP290*	c. 1429 C > T p.R477X^*N*^	c. 1429 C > T p.R477X^*N*^		+		+			
292	Pakistani	*CEP290*	c. 954delT p.S318fs16X^*N*^	c. 954delT p.S318fs16X*N*	+						
333	Pakistani	*CEP290*	c. 5744insT p.G1915FfsX1^*N*^	c. 5744insT p.G1915FfsX1^*N*^	+						
207	Pakistani	*RPGRIP1L*	c. 1945 C > T p.R649X^*N*^	c. 1945 C > T p.R649X^*N*^	+	+	+				small cerebellum
336	Pakistani	*RPGRIP1L*	c. 1945 C > T p.R649X^*N*^	c. 1945 C > T p.R649X^*N*^							diagnosed with MKS
158	Pakistani	*CC2D2A*	c. 3540delA p.R1180SfsX6^*N*^	c. 3540delA p.R1180SfsX6^*N*^	+	+	+	+	+		low set ears, pulmonary hypoplasia, intestinal malrotation, markedly enlarged pancreas- irregular ducts on histology, brain shows dilated fourth ventricle with small cerebellum, poorly developed pyramidal tracts and some possible dysplasia in the basal ganglia
180	Pakistani	*CC2D2A*	c. 3540delA p.R1180SfsX6^*N*^	c. 3540delA p.R1180SfsX6^*N*^	+	+	+			+	
261	Jordanian	*TMEM237*	c. 1066_1067dupC p.Q356PfsX23	c. 1066_1067dupC p.Q356PfsX23							meningomyelocele, developmental delay, cortical visual impairment
178	Pakistani/Mirpuri	*TMEM67*	c. 1615 C > T p.R549C^*N*^	not detected							diagnosed with MKS
16 + 17	Pakistani	*CC2D2A*	c. 685_687delGAA het p.E229del	not detected		+		+			
66 F1 + 66 F2	Pakistani	*CC2D2A*	c. 685_687delGAA het p.E229del	not detected	+	+	+	+			absent uterus, micrognathia, bilateral talipes, low set ears, wide spread eyes
**Non-Consanguineous**											
106	British	*MKS1*	c. 1408-35_1408-7del29	c. 1408-35_1408-7del29	+	+	+	+			
77172	Finnish	*MKS1*	c. 1408-35_1408-7del29	c. 811delC p.H271fsX29^*N*^							diagnosed with MKS
74699	British	*MKS1*	c. 1408-35_1408-7del29	c. 1408-35_1408-7del29							diagnosed with MKS
162 + 163	British	*TMEM216*	c. 253 C > T p.R85X^†^	c. 253 C > T p.R85X^†^	+	+	+	+	+		facial dysmorphism, postural deformities of limbs, small perimembranous ventricular septal defect, intestinal malrotation
176 + 177	British	*TMEM67*	c. 1426 C > T p.P476S^††^	c. 2440–3 C > A	+	+	+	+			flexion deformity of elbows and wrists, low set ears
186	British	*TMEM67*	c. 755 T > C p.M252T	c. 653 G > T p.R208X†††	+	+					
302	British	*TMEM67*	c.755 T > C p.M252T	c.651 + 5 G > A p.V217Vfs^*N*^		+		+		+	agenesis of corpus callosum
83527	Norwegian-Indian	*TMEM67*	c. 755 T > C p.M252T	c. 2882 C > A p.S961Y^*N*^	+	+		+			
74406a + b		*TMEM67*	c. 1351 C > T p.R451X	c. 2108 T > A p.V673A		+		+			mental retardation, retinal coloboma
210 + 239	Dutch	*CEP290*	c. 679_680delGA p.E227SfsX2	c. 1984 C > T p.Q662X		+		+		+	abnormal cerebellum, wide nasal bridge, extended abdomen*,* thoracic and abdominal *situs inversus*, intestinal rotation, small bladder, uterus duplex
153 + 154	French	*CEP290*^*a*^	c. 2251 C > T p.R751X	c. 4864insTdelCG p.R1622FfsX9^*N*^		+					
166	British	*RPGRIP1L*	c. 1829A > C p.H610P	c. 721_724delAATG p.N241fsX25	+	+	+			+	
128	British	*CC2D2A*	c. 3544 T > C p.W1182R	c. 3774_3774insT p.E1259fsX1							diagnosed with MKS
36 + 36A	Pakistani/Gujarati	*RPGRIP1L*	c. 466 C > T p.R156C*^N^	not detected	+	+					
111 + 112	Portuguese	*CEP290*	c. 1451delA p.K484fsX8	not detected	+	+	+				
202	British	*CC2D2A*	c. 685_687delGAA p.E229del**	not detected	+	+					craniofacial abnormalities related to oligohydramnios, bone-cartilage junctions showed disarray

CLP, cleft lip/palate; DPM, ductal plate malformation; DWM, Dandy-Walker malformation; OE, occipital encephalocele; PD, polydactyly; PK, polycystic kidneys; * *in cis* with c. 3790 G > A het p.D1264N, ** *in cis* with c. 3893 T > A p.V1298D; ^†^ p.R85X allele was present in 2/10,266 European/African/American controls in the Exome Variant Server (EVS) database, ^††^ p.P476S allele present in 6/7,012 European/American controls (EVS), ^†††^ p.R208X allele present in 8/7,012 European/American controls (EVS). The remaining changes are excluded in about 10,000 European/African/American controls (EVS). ^*N*^ indicates a novel mutation that has not previously been reported. ^*a*^ indicates that affected siblings 153 + 154 are compound heterozygotes for *CEP290* mutations, but both also carry a third heterozygous putative pathogenic mutation in *TMEM216* c. 188 T > G p.L63R.

In addition, six families were identified with a single heterozygous mutation in an MKS gene, but we were unable to identify a second pathogenic variant. *CC2D2A* p.E229del is probably a common variant, and it was detected as a single heterozygous variant in two families of Pakistani origin. In family 36 + 36A and family 202, two changes in the same gene were detected but were inherited *in cis* from the paternal line, so the pathogenic potential of these variants is unclear. We did not detect any other potential pathogenic changes in any of the seven MKS genes that we screened for these patients. Family 178 has the single heterozygous missense mutation p.R549C that is likely to be pathogenic because the same mutation is found in the homozygous state in family 205. This mutation may be a Chinese founder mutation (K. Szymanska, personal communication).

We identified homozygous mutations predicted to be pathogenic in 50% of consanguineous families (Figure 1b). Two families had mutations in *MKS1*. Family 264 had the homozygous *MKS1* mutation c. 1408-35_1408-6del30 (Figure 1c), which is almost identical to the “Finn major” Finnish founder mutation (c. 1408-35_1408-7del29) with one base-pair difference. Since family 264 is of Jordanian origin and, therefore, has a different genetic background from northern European patients with the “Finn major” mutation, this finding suggests a mutation hot spot in this intronic region of the *MKS1* gene. We identified three different homozygous mutations in *CEP290* in Pakistani families. Two of these were frameshift mutations with one nonsense, predicted to cause nonsense-mediated decay.

The majority of identified mutations were found in *TMEM67* (Figure 1b), comprising n = 14/47 (29.8%) families. Two splice-site mutations and one missense mutation were identified as recurrent in families and, therefore, probable founder mutations (Figure 1d). In patients of Pakistani ethnic origin, the two *TMEM67* splice-site mutations are c. 1546 + 1 G > A and c. 870-2A > G, previously reported as common mutations in *TMEM67*[6]. We identified the homozygous missense mutation p.R441C in two families (319 and 347), a mutation reported previously in the heterozygous state for patients with COACH syndrome [7]. A missense mutation affecting the same amino-acid residue, p.R441L, has also been reported previously in an MKS patient [8]. Since families 319 and 347 have different ethnic origins (British and Pakistani, respectively), this emphasizes the mutability of arginine residues and their importance to the function of the protein since the neighboring residue p.R440 is also mutated in MKS and MKS-associated ciliopathies [9-11].

We identified probable founder mutations in both *RPGRIP1L* and *CC2D2A* for families of Pakistani ethnic origin. The *RPGRIP1L* nonsense mutation c. 1945 C > T p.R649X was observed in families 207 and 336, which are reported to be unrelated. The frameshift mutation in *CC2D2A* c. 3540delA p.R1180SfsX6 occurred in the unrelated families 158 and 180, with polydactyly noted as an obligatory feature in all affected individuals.

Two-thirds (n = 13/19) of non-consanguineous families had their causative mutations identified (Figure 1e), with the majority of mutations (n = 10/13) in the compound heterozygous state. The majority of identified mutations were found in the *TMEM67* gene, with mutations in *MKS1* and *CEP290* the next frequent. In our cohort, the “Finn major” mutation was found in all *MKS1-*mutated patients, either in the homozygous state for two patients (families 106 and 74,699 of British origin), and in one patient (family 77,172 of Finnish origin) as a compound heterozygous mutation *in trans* with the frameshift mutation p.H271*fs*X29. Overall, the *MKS1* “Finn major” mutation was the most frequent (Figure 1c). The heterozygous missense mutation p.M252T accounted for 30% of identified alleles in *TMEM67* in non-consanguineous patients. The previously reported common Finnish *CC2D2A* mutation is absent in our cohort [12], even though the *MKS1* “Finn major” Finnish mutation seems frequent. This suggests that the latter is more widespread throughout European populations, whereas the *CC2D2A* mutation seems to be less common outside Scandinavia.

## Discussion

There are previous reports of genotype-phenotype correlations in MKS [8,9,13]. We confirm some of these correlations with the available clinical data for our cohort of MKS patients. Occipital encephalocele and polycystic kidneys were almost obligatory features for all patients. Individuals with *TMEM67* mutations frequently had a diagnosis of ductal plate malformation in the liver (n = 10/19), but polydactyly was infrequent (n = 3/19) compared to *RPGRIP1L* and *CC2D2A* mutated individuals (n = 4/6; *P* < 0.001, chi-squared test) The Dandy-Walker malformation (or a posterior fossa defect) was occasionally observed in patients with *TMEM67* mutations (n = 3/19). Retinal colobomata were only observed for *TMEM67*-mutated individuals (n = 2/19). Furthermore, *situs* or gut malrotation defects were never caused by *TMEM67* mutations (n = 0/19), in contrast to the occasional manifestation of these clinical features with *MKS1*, *TMEM216*, *CEP290* or *RPGRIP1L* mutations (n = 4/17; *P* < 0.05, chi-squared test).

Mutation analysis in our MKS and MKS-associated ciliopathy cohort has allowed us to observe some common mutations that have arisen from probable founder effects, supported by the observation of common shared haplotypes in affected individuals (Additional file 3: Figure S1). These observations will allow initial prioritization of gene and exon screened in affected patients. Patients diagnosed with MKS, and that have additional features of ductal plate malformation and/or retinal coloboma, should be tested for *TMEM67* mutations since, in any case, MKS mutations are most frequent in this gene. In consanguineous families of Pakistani origin, the *TMEM67* splice-site mutations c. 1546 + 1 G > A and c. 870-2A > G should be prioritized. In addition, screening for missense mutations between amino acid residues 250 to 570 would detect a third (n = 10/29) of all of the *TMEM67* mutations in this cohort. It is likely that missense or nonsense mutations of conserved arginine residues in this region (for example, R441C, R451X and R549C), may be recurrent and could, therefore, be founder mutations in other population groups. In consanguineous Pakistani families, the probable founder mutations *RPGRIP1L* c. 1945 C > T p.R649X and *CC2D2A* c. 3540delA p.R1180SfsX6 should also be prioritized. For families of northern European (including British) origin, without a known history of consanguinity, testing the *TMEM67* missense mutation p.M252T may be useful, but the most common mutation is the *MKS1* “Finn major” mutation. Our results demonstrate the broad phenotypic variability in MKS and the lack of clear genotype-phenotype correlations to guide diagnostic choices. Furthermore, some MKS mutations, such as the *TMEM67* p.R440Q missense mutation, are allelic for Joubert syndrome and other ciliopathies.

The molecular basis of the phenotypic variability in MKS may arise from oligogenic inheritance [14], where a third modifier allele modifies the phenotypic effect of two recessive alleles. It is interesting to note that many ciliopathy and ciliary-related proteins interact and are reported to create functional modules that are localized to discrete structural regions of the cilium, such as the transition zone [1-3,5]. The effect of modifier alleles may be to abrogate interactions between components of a functional module, which may disrupt protein complexes or signaling pathways giving rise to the ciliopathy phenotype. We identified four different heterozygous changes in six patients, in the absence of a second detectable pathogenic mutation in the same gene or any other mutations in other MKS genes. These heterozygous alleles could be potential modifier alleles, but we have not exhaustively excluded the possibility that a second pathogenic mutation is a large deletion spanning exons and/or introns in the same MKS gene, which would not be detected with PCR amplification and direct sequencing alone. Although we have not assessed gene dosage in these genes by, for example, a multiplex ligation-dependent probe amplification strategy, we have seen no evidence of allele drop-out at the same or other known MKS loci following genome-wide SNP genotyping of consanguineous patients (individuals 178, 16 + 17 and 66 F1 + 66 F2). We have also not excluded the possibility that a second point mutation occurs deep within introns or regulatory elements of the same MKS gene. Interestingly, two affected siblings (153 + 154) were compound heterozygotes for *CEP290* mutations (Table 2), but also carried a third heterozygous putative mutation c. 188 T > G p.L63R in *TMEM216*. This was the only occurrence of possible triallelic inheritance in our cohort for the seven MKS genes that we screened, although the pathogenic potential of this third *TMEM216* allele remains unclear.

## Conclusion

In conclusion, our data provide further useful information about the mutational load in MKS patients from different ethnic backgrounds. With the ever-increasing power and affordability of genetic sequencing technologies, there is now the clear opportunity for the further rapid and robust identification of mutations in patients referred for a defined condition. As a prerequisite, there remains a pressing clinical need for the dissemination of mutations identified on a research basis, and the establishment of databases that provide detailed clinical phenotypes and allelic series for specific genes.

## Abbreviations

BBS: Bardet-Biedl syndrome; JBTS: Joubert syndrome; LCA: Leber congenital amaurousis; MKS: Meckel-Gruber syndrome; NPHP: Nephronophthisis; SLSN: Senior-Løken syndrome.

## Competing interests

The authors declare that they have no competing interests.

## Authors’ contributions

HJ, YR, SM-S, BC, CB and RC referred patients for research studies and collated information on phenotypes. IB, HL, HJ and RC prepared DNA samples and collated information on phenotypes. KS, IB, CVL, SC, HL and CAJ screened MKS genes. KS, IB and CAJ contributed equally to the preparation of the manuscript. All authors read and approved the final manuscript.

## Supplementary Material

Additional file 1 ** Table S1.** Primer sequences of microsatellite markers used for genotyping of MKS loci. Click here for file

Additional file 2 ** Table S2.** Primer sequences used for direct Sanger sequencing of MKS genes. Click here for file

Additional file 3 ** Figure S1.** Haplotypes for common mutations in *TMEM67*, *CC2D2A* and *RPGRIP1L*. Putative shared common disease haplotypes (genotypes in bold) are shown for the indicated microsatellite markers on the left that flank the MKS genes *TMEM67*, *CC2D2A* and *RPGRIP1L*. The numerical identifier of each affected individual is shown underneath each haplotype (see Table 2 for further details). Click here for file
