# Supplementary material for: Founder mutations and genotype-phenotype correlations in Meckel-Gruber syndrome and associated ciliopathies
Source: Cilia. 2012 Oct 1;1:18. doi: 10.1186/2046-2530-1-18 (PMC3579735; doi:10.1186/2046-2530-1-18)
Supplement: Additional file 1 — Table S1. Primer sequences of microsatellite markers used for genotyping of MKS loci. [file 2046-2530-1-18-S1.pdf]

GENE	MARKER	FORWARD PRIMER (WITH 'FAM' DYE)	REVERSE PRIMER
MKS1	D17S1606	TGGTATTCAATCCTGGAGC	TGATGAGTCTTCATAGCCCC
	D17S1290	GCCAACAGAGCAAGACTGTC	GGAAACAGTTAAATGGCCAA
TMEM216/TMEM138	D11S4191	GCAAGATGGCCAATTAGAAG	TTTTGGTTGGAATGTAGTTGTTTAT
	D11S4076	CATGAATGCTCTTGTCCC	AACCCCCTGGAAAATAGACT
TMEM67	D8S1988	CCTTTGGACTCAGACCAGAA	TAGTCAGAGTCCTCAGAGAAACA
	D8S1699	CAACCTGACCCTGCCA	CATGATGTTCTAAGCATATCTGC
CEP290	D12S1719	TCCTCCAGTTTCAGTAATGTTT	GGTGGTTGATGCCTGTAA
	D12S1710	AGGTTTCTGGGTTCCTGATA	CCATAATCCGTAGGAGCAA
RPGRIP1L	D16S3034	TAATCTAGTTAAAGATGCAACTGCC	GCTCAGAAGTTTTGATGCC
	D16S771	GTCCAAAACACCACCCTCTA	AAGTAGATCAGTCATCTTGCTGC
CC2D2A	D4S1511	AGCCTCTGTAATCTTGTGTG	TCCATTACTCAGGGCTCTC
	D12S2960	AAGGCTTTATCATTAAGAATCCTA	TGAGGGTATAGTTACCATCTTTT
TMEM237	D2S2309	TGTCAGGCACTTCGCTA	TGCTTCTTATTGTACCCAAA
	D2S1384	AATAGAGGGCCCTTGCTTAA	TTTGGGATAAAAGGTATTTTGC
