# Supplementary material for: Founder mutations and genotype-phenotype correlations in Meckel-Gruber syndrome and associated ciliopathies
Source: Cilia. 2012 Oct 1;1:18. doi: 10.1186/2046-2530-1-18 (PMC3579735; doi:10.1186/2046-2530-1-18)
Supplement: Additional file 2 — Table S2. Primer sequences used for direct Sanger sequencing of MKS genes. [file 2046-2530-1-18-S2.pdf]

Exon	Forward	Reverse
	MKS1	
1	CCGTTGCCAGGAAAACAG	AAGCAGAAAGTGGGGACCTC
2	CTTGGCAACCCTACCTCCTC	TGCCGGGCAACTCTATAAC
3	CCACAGAACAAGAAGTGGTGC	CCAGCCATGTGGAGGTAGAC
4	AAAACATGTCAGATAGCATCAGTTC	AGACAGGCTACTTGTCTCCCC
5	TCTTCTTTGGGTTGTTTGGC	TCCCTTCCCTCTCTCTGAGG
6	GAAGTGGCTCTCCACTGGG	GCTGGTGAGAGGAGAAAAGG
7	CCTGCAGTGGGCTCAGTATC	AAGTGCAACCAAGGGAAGG
8	CTGGGCTGGGAGCTATCC	CAGCTCCAGGCACTGAGG
9	TGAACTTTGGTCTAGCTTGGG	CCACAGTCAGAATGCTCCG
10	GATTTGTAGATGGGGCTGGA	AGCAGACAAGGCCTCTACCA
11	TTGGGTGCTAAGGGTGGTAG	AGCCTGACCAAATGCCTCTA
12	CTCTCCGAGAAACACCCAAG	CCACACACAAGTCACCCAAC
13	TTGGGTGTCCTTTTGCATCT	TCTGGGAGCAAGGAGAGAAG
14	AGTGCTGTCTCTTGGTGAAGG	CCAGAAGCATTCGAGTGTTAG
15	GTCATTGCTGGGGAGTCAG	CACCTCCTACAGAAGGACAGG
16-17	GGGGGTGTCTAGAGAGAGCA	GCACTGCAGAGAGAAACAGG
18	CATGGGCCTGTTTCTCTCTG	CCACAGGGTCTCTGGCTTTA
	TMEM216	
1	CGTCGCTCACACCTTTTCTT	GGGTTTAGGTCAAGCTCTGG
2	CAGCGTCTAGTTTCCCCAAA	GGGCAGAAAAGAACGGAAAA
3	GCTGAGACTGATGTCCTCTTTTT	CAGATCCAGTCTATGATAGTCAACC
4	TTGCCATTCCATCACATCTC	CCCCTTAGGCTTCAGAAGAA
5a	TATCCTTTGGGGCCAGGAG	ACAGCTCGTACAGGGAGCAT
5b	CCCCTGCTGACCTCAAGTT	GCTCCTGAGAACAAACTGCTG
	TMEM138	
1	TGCAACTGGAGTGGTACCTG	GTCCTGGGATTATGGGTGTG
2	ACTTTGCCCTCAGCATCTCC	TGCCTGTAATTCCAGCACTTT
	TMEM67	
1	GGAGGCTGTGAGGCTTCC	GGTGAAACTCCATTTACGGG
2	TTATTAAAAGTTTCACTATCTGGGAAC	AATTTTAAAGTCTGTGGGGAAAC
3	CATACTCTTATGGCATTTTGAACTTAC	AGAATTGCTTGAGCCACTGC
4	TTTATTATGGAGATACTTGTTATGCC	TGGGCCTCAGCTTCTGTATC
5	CTAATCCATTGTTTAGAAAGACAGC	CCTGAAATTACTAATGGATGAAATG
6	TTCAGTTGCTTATGCCTTTTCA	CAAAAGGGTTTCTCATTCTAGCC
7	TGTGAGACATTTCCCATTCAAC	GCCCACAAGAAAGAGTTTATTC
8	TGGAACAGACCTGCACAAAG	GCCAAGGAAGATTCTGTCTCC
9	CTGCTGGACTGAGCACTGTT	CAATCCAGAAGTATCCACATATTCA
10	AAAACCAGTCTAATTCTTTCAGGTC	CTCTTGGCTTTGTCTCAGGC
11	TATCCATGTTCGGGTTTGAG	GCAACAAGAGCAAAACTCCATC
12	TGCTTGCTAATTTTCAATTGTG	CAACAGTTTCTAAGTCCACCAGC
13	GAACGCCATTGTCAGGTG	CCGACCAACTGTTCCAATTC
14	TGTGTAACCTTCCTTAGTCCTTG	TCAGGATCCATAAGAACTCACC
15	TGGTAAAACCCAGCTACAAATG	TCTGGGGAAAGAATAGCTGG
16	GAGCACTTCCTTACTTGTTCACAG	TGAGAAGGATCCAGAATGGTC
17-18	GGCTTCAGGCTTAAGAAATGG	TGCTTTCTTTCAAAACCAAAGTG
19-20	TCCTTAGTGGTATTATAACTTCATGTG	TGGCTTCACAACAATATTTCAAC
21	GGTGAGTAGGGAGAGGTTTTCTG	TGCTACAGAAAGAAGGATGTGG
22	GGAACTGTTAAAGGCCACTG	ACAACCAGTGGAAAATTGGC

23	TTGCCCAGACTTGTTTCTCC	CACTTGAACCCAGAAGGCAG
24-25	TGAATTTCACTTAGCACCTGAATC	AGGGAAGGTCTTAAGGTGGG
26	TCCTGCTGATTTTACATTAATTGC	TTGGGAAGCTGAGGCAAG
27	TTGTCACCAGAAGTTTATCACAGAC	TATTCCTTCTTTTGGTGGGG
28	TGTATCACTATGGGATTCAGATACC	TGCATTAAATACTACACACAATGGG
	CEP290	
2	ACCAATAATACTGTGTACCTTG	CAGATTGTGACAATTATAGTTG
3	CAACTATAATTGTCACAATCTG	GTTCCACTAATAGCCAAACC
4	GTGCTTACATTCCAGTATAAAG	GTTTAATGAACAAATGGAATTCA
5	ACCTTATAATCATGATGGACTC	AATAACCATGATTACAATCATCC
6	TTGTTGACTCATTTGAACCTC	AAAAAGCCAGGTAACTTGAAC
7	ACTGCTGAATTTTATCTTCTTC	TTAGAAGACTCCAGTCCTGG
8-9	CAAGATAATATGCATCATTTTCCC	ATGAAATTAAAGTTTTTAGGAACC
10	AGAGGACACTTATGGCTGCG	GTAATGAGATAATATGAAGTCTG
11	CACATATGTAATGTAATGTATCC	CTAATAAACGTGTTATAAACCAG
12	GTATCATAAATCTACTAACGGTG	ATCGTTCAGAGTTCCAACTG
13	CTTGTACCCACAAGAAAATATG	AGAAAACTCAATATTGACTTGAC
14	TGATTTGAAGGAATAAGTAGTC	CTGTGAATGGCAAGAATAATTC
15	GTACATTTTCCTTTAGACTTAG	ACTTGTAAATCAGGTTGCGC
16-17	CATTTTTGCAGCTTATTTGAATG	ATATCCAGACAACTCACTTATC
18	ATTAAAGTGTTGGAATAGTAGG	TATTTTCCTTTACTCTCTTTGC
19	ATTGATCAAACTTTTCTTAACTTG	ACAGAGGTAATTAGGAGTAAAG
20	CCAATGATGTCTTTGGTATATG	AAATATCTCATCAGAAACTATGG
21	GTCCATTTTATTTAAAGACAGAC	TTAATTCAAGGGGCATTTTCTC
22	TATGGTTGAGGTAAAATTCCTG	AGTACTATCTGCATGCTTTGG
23	TAACTTTCCTATAATGTTGTCAG	TAAGTTCCTAACAGTAGTTACC
24	ATACCTCTTGTGTTGAGAAAAC	CACAAAGACACATCCATATTAC
25	TATGCAATATTGTACAAAGTAGG	TGATACCATCCTATCTTCTGC
26	AAAGTGGCTAGTGCTTGACC	TGTTAAATTTATATAAATGCAGGC
27	AACTGGATTGTGAGTTTTAAGG	AGGATTATTCATCTGCCTAAG
28	ACAGCATCTAAAATATCTGAGG	AGATCCAGACAAACCACTTAAC
29	AAGGCCAAGTAAAGAGGATTG	TACTACTAAGAATTGTATACCTG
30	TAGAAAGTGTACTTAATTGTTCC	CCCACTCCCAACATCTAATG
31	CATTTGGATTTTAAGTTTGTTTGA	AGCCCAGGAGTTCCAGCTAT
32	CATTATCATCAATGGAGGAATG	TAGTCATTTGTGCAATATTCTTG
33	CCTGTTATGTGCCTGATGTC	TGAGTTAACACTCTAGACTATG
34	ATCTATGTTTTATCATACAGCTG	ATCATTCTATGCATTGCCCTC
35	GCATTTTAAAGGGAAAAAGATAC	CACTTTAGGGTAAAATAATATTTAG
36	ATATGGAGATACTGTTTCTTCC	GCTGAATTTTAATTTACATGGTC
37	AATATGGAATAAGTATGGCATTG	AGCAAACACTTATGTTTATCTTC
38	GTGACAGAGTGAGACTGGG	ACAACACGGAGATTTATACTAC
39	ATAGTAGGAAGTAATAAAGCTTG	TAGTGAATTCTCTTCCAATAGG
40	GTTCCTTTTATCATTGATACTTC	AAGTAGAAATAAACTACTACCTC
41	GTGATAGCTTCAGAAAGTTGC	CAGAATTAATACAGCCAGGTC
42	AACATATTTACATATTCTCTAGG	TAAAGCTATATAATTTCCAGGTC
43	TTTGGTTTGGTAATGAGTATGC	TTCAATTTCTAGGGGTCAACC
44	CATGGAGGGTCTTGTAGGTCA	GGCCAGATTAAGAAAGAAGTTGAT
45	TATCCAGTATGTCTTTTATGGC	ACCATCACCATGATATATTAGG
46	TTTGCCTTTTCTTTTCAATGGC	TATCTAAACTTTTCATTTCTGGC
47	TGTTGTATTGTTGGTACTTCG	TTAGCCTTGCCTCTCATAAG

48	TGGTTTCTAAAACTACTTTGAAG	ACTTCCAGTTTTTCCAAGAGG
49	TAGAGCCCCAGGTTATTTTTG	TGTTCATCAGGAAGAAACCAG
50	TTAGTACAGTTATTTGAACTGAC	ACAATGCAAGGAACATCTTGC
51	ACGCTTTGTTAAAAATGTGTATC	ATGCTTGTCTCTAGTTGTAGC
52	TCACTAGTTCATAAGAAATGCC	AATTCGATTTTACAGGGAGAC
53	CCATTACCTTGAACTCATTCG	TAGGATACGTAGTTAAAGATGG
54	ATTCAGGAATACTTTGGCTTTC	TTCGGAGAACTGCTTATTTCC
	RPGRIP1L	
1	TTTGGTTCATTCCATTGCATAG	ACTTAAATGGTTTGGTTTGTATGAG
2	AATGGATACTATTGTTATTCAAATTCC	CATCCTGCCTTGTTCCAAAC
3	TCTTCACAGTGATTATGTTCCTTTC	TTTCTCTTCCTAAAGACACTTAAAAGC
4	TGGCTGAGATCACATATTCCC	GCTGTATTTCAGTGGAGCAAAC
5	ATTTCACTGTGTGCAGAGGC	GCAGTTAGTAGGTCGAGCATAGG
6	CCATAGATTTTCTTCTAACTTGCG	AACAACTTGAATACTACTTTTGAATCC
7	GGTGAACTTCGGGAGTAAATTC	TTTGCTGTGCTCAAAACACC
8	GCAGCTACCATTTAGCAATCG	TCTGACAACAGCAAATGCTTAC
9	GGTCTCTGCGCCACTAAAAG	TGACTGATTCAATGAAGAAACATTAG
10-11	TTTGAAATGTGTCCTTAAACCTG	AAGGGTTACAGATAAGGGTCATC
12	TCCATCAGGAATGAAGCAGTC	GGGTGACAACAGCAAAACTC
13	TGCTTGAATCATAGTTGCTTTG	CACTGTAAGTGCAGATTTGGATG
14	TTCAAAAGCCAGTTTTCATTTTC	GTCCTTGCTCTAAAGGCACC
15	CCAATCTCACATCATTTGTGC	AAAAGACACTTGATGGCTGTG
16	TATCTTATTTGGGGCTTGGC	AAAGACTGAGAAGAGGTTAGGGTG
17-18	TTCACATGGTAGAGCATGAGTC	TGTTTCCATTTAAATCAGGTAGGG
19	TTGAACATTTGCATTGCTCTC	GGGAAATGCTATGACTTCCTG
20	TTTACATGCTTTATTATTCAACTGTTC	AACAGGTATCGTGAAGTAGACGC
21	TTTTCCCTAAAATGTAATGTGTCTG	TGTACTGTGAATGAAAGGCAGC
22	TGACATGCTGAGATGAAGTTGTC	CGGATCATGAGGTCGGG
23	TTCAAGAAAAGAAGCCCATTG	TTCTTAATCTTTATGCTACTGGTTTG
24	GTGAGGTGGCATGACTTGTG	TGCCTTTTATTTTAACCATGTCC
25	GTTTTGTCTCGTGGTGATTTC	AGCACCCTTGGAGCCAG
26	CCCAAGATGCCAGTGAACTT	GAGGGGTGGCAGTTATGAGA
	CC2D2A	
1	GGTTCCTGGTGAGTGGAAAG	GTGCAATAGATGCTGGTTGG
2	GATGATGAGAGCCGCACAG	GTATGGACCCCCGCCTTATT
3	ATAAGGCGGGGGTCCATACT	CTCAAAATGGCGCTTCTCTC
4	TCAGGTGACAGAGACTGTGGTT	TGGGGTAAAGTGGGTGGAG
5	TCCAAATCTGAAATGTGACCTTC	TGTTAGTCCATTTTGCATTGCT
6	TAAATTACCCAGTCTCAGATATTCC	CCTTCAGAGCCATGAGAAAA
7	GGCAGGACTGAGGTTTTGTC	TGTGCCAATGCAAAATAAGA
8	TTTCCTTGCTCTTCCCCTTA	CTTGGACTCAAGCGATCCTC
9	GAAGATAAGCCTTTGGGATGG	ACACCCGGCCTGTAGTCA
10	CAACTGCCTGTGGCAGATATT	CGTGGAGTGTCATTCTCCTG
11	CATGAGTTTGGTATTGTGAATTATTTT	GGAATCAGTTCTGGAGATGAGG
12	TCATCTTTTCCTTGGTTCTGC	TGGGTTTTGAGCAGTTGAGA
13	GGGGGAGGAAGTACATGAAA	GCCTGATGTTTGTTCTTTATGTGT
14	TGGCCTCTAAAACCTGGAAA	CACACATCCTAAACCGAGGA
15	CACCATTTTGCCCAGGAG	TGACCATTTCCTTACATCCTCA
16	TCTGCCTAAAGAGAACAAAATCAA	CCTGGGAAGTGGTTACATGC
17	AGCATCTGCCTCACAGGATT	AGCCAGTCAATTCATTCATCC

18	AGGCACAGAGAGGTCAGGTG	CCCATGTCGTGCATGTGT
19	TGTGCCCTTTTCAGTGTCTG	GTCTCGAACTCCTGGCCTTA
20	TTAGGGCTGGAACAGTGACC	TGGTTGTCTGGGAAGTGAAA
21	CACCCACTCCAAGTTCCATT	TGGCACCTGACAATAAAAGG
22	GCTTCCTCAAGTCATGCTGTC	ATAGCCAGGCAAGAAGCACA
23	AGGGAAGCATGAACACCAAA	CCGCTATTGCTAGTGACAACA
24	AGCCACCTGTCTCAGAATCC	TGGCTTGCATAAGCTCACAT
25	GGGGGACACTGAGATGACA	CATTATTCCTGGCCCAAATG
26	GAGTTTTTCAGGGGCGTGT	AACCACGTTAAGAATGAGAGTGA
27	GGCGACAGAGTGAGACCCTA	CTTCACCTTAGCCGTTTCTCA
28	TTTGGGTATTTTTCAAATGCTG	GATCGGGACCCCTTTCTG
29	AATCTTGCTGCTTTCCTGCT	AGGCTCATCTCCAACTCCTG
30	CCTTTCTGGCGAGTGCTTAG	TTGCTGATTGACCCGATTACT
31	TCCCAAACCATACATTTCCTG	AGTCATCTGCCCGCCTTG
32	AGGATGTTTGGTGAGGATGG	TTTTTATGGCTAATGAATGTTGC
33	GAGAAAACACCATGCCCACT	CCTGAGGACCACTGAAAACC
34	TGCAAAACTAAGCATTCGACTT	GCTTTGTCATTCTGCTTCTCG
35	TGCGGCCTATATGAACACTT	CTGTTTCTTGTTGTGAGTTGAGC
36	CTTTTGGTGGGTCATGGGTA	AAGTTGCCTAAATTGTTTCACATTC
37	CAATCATCTGAATCCCCACT	TGAAAAATGGTGGTATTTTGCT
38	GCTGAGTAGAAAAACACAAGCAAA	TTGCCCAAGCAAAAGGTAAG
	TMEM237	
1	ACACCCAGGCCTGCACAGACTCG	AGATGAGTGAGGCGTCGTCGTG
2	TGCACGCCTCTCACCGCAACAT	TTCCACCCACCCAGAGACCCAACC
3	GATGGTATTTGAAGTAAAGAT	GAGTTTCCTGAATCATTGTA
4	TGCACGCCTCTCACCGCAACAT	GGTGCAGTCCTCATTATAG
5	TCTTCCCCTAATCTATAAACG	ACTGGCCTTAATTCTATCCTC
6	TGAAAGAGATGGGGTTATTC	GCTTAAGCCATTGGAGTTAT
7	AAAACATTTTTGCCCAATTA	CAAACAAAGCATCAGGGTAA
8	GCCCTCAGAAATGTAAGTA	TCAGATTTCCAGAAGTATAAAG
9	TCGCTAGTTGTTAGTAATTACG	TACCTACCATTTTAAGTTGT
10	TAAACATCCTATTGGAATTG	AAAGAACAGAAGTAGACTGGC
11	CATGCGTGTTAAGTATACACT	AGGTAATGTTTATAAGGAGC
12	GGATGTCTGGCCTATTGAAA	GCAGGTGGACATAACAATAG
13	TTTCTCTAGGAGAGGACTGCT	TTGGCATTCTGTTACACC
14	CTTGAGGGCTTTCTATTAGT	CTTCTACCATATTCGCTAAAT
