# Supplementary figures and images for: Founder mutations and genotype-phenotype correlations in Meckel-Gruber syndrome and associated ciliopathies
Source: Cilia. 2012 Oct 1;1:18. doi: 10.1186/2046-2530-1-18 (PMC3579735; doi:10.1186/2046-2530-1-18)

TMEM67

D8S1988 289 287 289 285  
D8S1699 215 217 215 221

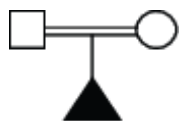

D8S1988 289 289  
D8S1699 215 215

29A

289 285 289 285  
215 207 215 221

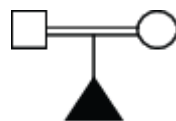

289 289  
215 215

33A

289 285 289 285  
215 225 215 217

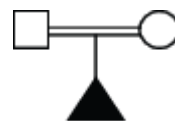

289 289  
215 215

70

285 285 289 285  
215 225 215 215

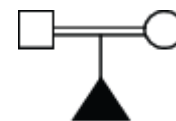

285 289  
215 215

76

CC2D2A

D4S1511 150 150  
D4S2960 245 245

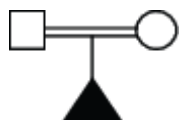

158

150 150  
245 245

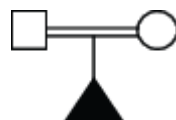

180

RPGRIP1L

D16S3034 275 275  
D16S771 257 257

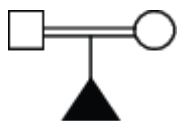

207

275 275  
257 257

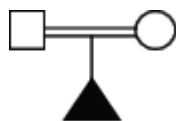

336

Supplement: Additional file 3 — Figure S1. Haplotypes for common mutations in TMEM67, CC2D2A and RPGRIP1L. Putative shared common disease haplotypes (genotypes in bold) are shown for the indicated microsatellite markers on the left that flank the MKS genes TMEM67, CC2D2A and RPGRIP1L. The numerical identifier of each affected individual is shown underneath each haplotype (see Table 2 for further details). [file 2046-2530-1-18-S3.pdf]
